# Effect of citrus waste on blood parameters of broiler birds with and without cocktail of enzymes

**DOI:** 10.14202/vetworld.2019.483-488

**Published:** 2019-04-02

**Authors:** Devi Prasad Behera, Amrit Pal Singh Sethi, Chanchal Singh, Udeybir Singh, Manju Wadhwa

**Affiliations:** 1 Department of Animal Nutrition, Guru Angad Dev Veterinary and Animal Sciences University, Ludhiana, Punjab, India; 2 Department of Veterinary Biochemistry, Guru Angad Dev Veterinary and Animal Sciences University, Ludhiana, Punjab, India

**Keywords:** antioxidant levels, citrus waste, cocktail of enzymes, lipid profile, liver and kidney function test

## Abstract

**Aim:**

This study aimed to assess the effect of different levels of citrus waste (CW) with or without enzyme cocktail on blood profile of broilers.

**Materials and Methods:**

CW was sun-dried and grounded to powder CW. 256-day-old birds were distributed into eight groups; control (C), CW-supplemented diets (2.5% CW, 5.0% CW, and 7.5% CW), enzyme (E) cocktail supplemented diets (CE, 2.5% CWE, 5.0% CWE, and 7.5% CWE). The diets were fed during starter (0-14 days), grower (15-21 days), and finisher (22-42 days) phases. Blood was collected from the wing vein from four birds per treatment. Serum was separated out after centrifugation and stored at −20°C until further analysis. The samples were analyzed for liver function test (glucose, total protein [TP], albumin [ALB], and globulin), lipid profile (cholesterol and triglyceride), kidney function test (alanine aminotransferase, aspartate aminotransferase [AST], blood urea nitrogen [BUN], and creatinine), and antioxidant levels (catalase, superoxide dismutase [SOD], lipid peroxidation [LPx], glutathione peroxidase [GPx], glutathione [GSH], and Vitamins E and C).

**Results:**

Blood profile data revealed that supplementation of CW showed no effect on TP, ALB, globulin, and BUN levels. Plasma cholesterol, triglyceride, and AST levels decreased linearly with an increase in the levels of CW in the diet. Catalase and SOD activity increased non-significantly with an increase in inclusion level of CW in the diets. LPx, GPx, and GSH activities decreased (p≤0.05) up to 5% CW-fed groups. Vitamin E and C activity were found to be highest (p≤0.05) in birds fed with diet supplemented with 5% CW. GPx and GSH activities and serum Vitamin C levels were observed to be highest (p<0.05) in birds fed CW (at 5%)-based diet supplemented with enzymes.

**Conclusion:**

The blood profile showed that supplementation of CW up to 5% decreased cholesterol, triglyceride, and AST levels and improved the antioxidant status. Vitamin C levels were observed to be highest (p<0.05) in birds fed CW (at 5%)-based diet supplemented with enzymes.

## Introduction

The exponential growth of broiler industry has raised the demand for feed and feed ingredients. Poultry industry requires 10.9 million MT of broiler feed to satisfy the nutritional requirement of the birds, in the poultry production system; feed alone costs about 60-70 % of the total cost of production [1]. Increase in the prices and unpredictable availability of feed ingredients has a direct impact on the broiler industry. To meet the increased demand for feed, search for novel feed resources, particularly those not competing with human food, is a key for sustainable development of the poultry industry. The food processing sector generates approximately 1.81 million tonnes of fruit and vegetable wastes in India which are either composted or dumped in landfills or rivers, causing environmental hazards [2]. These wastes left after processing are rich in essential nutrients that have the potential to be used as feed supplement.

Citrus waste (CW), a by-product of citrus processing industry, is available in huge quantities in India which includes 60-65% peel, 30-35% internal tissues, and up to 10% seeds which constitutes 50% of processed citrus [3]. The term citrus covers oranges, sweet lemon/lime, lemon, kinnow, grapefruit, tangerine, etc. The major by-products of processed citrus are dried pulp, molasses, washed pulp solids, and essential oils. Dried citrus pulp contains almost 5-10% crude protein, 6.2% ether extract, 10-40% soluble fiber (pectins), 54% water-soluble sugars, 1-2% calcium, and 0.1% phosphorus [2]. Besides, citrus pulp contains active antioxidants including a mix of flavonoids, isoflavones, flavones, anthocyanins, coumarins, lignans, catechins, and epicatechins [4]. Flavonoids have been reported to decrease the blood cholesterol and also quench the free radicals, thereby exerting antioxidant activity in laying hens [5]. It was found that utilization of dried citrus pulp up to 16% in diet significantly increased serum glucose and high-density lipoprotein and reduced cholesterol, low-density lipoprotein, and triglycerides in laying hens [6]. The use of CW will not only be a cost-effective alternative feed ingredient for economical broiler production but also will help in preventing environmental pollution and maintaining healthy blood profile of birds.

The present study was conducted to assess the impact of supplementation of CW on blood parameters of broiler birds without and with cocktail of enzymes.

## Materials and Methods

### Ethical approval

All procedures used in this study were approved by the Guru Angad Dev Veterinary and Animal Sciences University and Committee (IAEC-CPCSEA, New Delhi).

### CW procurement

CW was procured from Punjab Agro Juices Ltd. (India). The material was sun-dried and grounded for further use. CW and other feed ingredients used were analyzed for proximate principles, phosphorus [7], and calcium content [8].

### Birds

Broiler chicks were procured from Guru Angad Dev Veterinary and Animal Sciences University hatchery. 256-day-old birds were divided into eight groups (quadruplicate group of broiler chicks having eight birds in each replicate) and were fed diet control (C), 2.5% CW, 5.0% CW, 7.5% CW, CE, 2.5% CWE, 5.0% CWE, and 7.5% CWE. The cocktail of enzyme (E) had β-glucanase, xylanase, pectinase, cellulase, acid protease, natural protease, mannanase, α-glucosidase, amylase, lipase, phytase, and α-galactosidase. Eight experimental rations were formulated [9] for each phase, i.e., starter, grower, and finisher (up to 42^nd^ day) on deep litter system.

Environmental condition of house was optimum for rearing broilers, i.e., temperature was maintained at 85°F with relative humidity between 30% and 40%. 24-h light was provided throughout the experimental period, i.e., light was hanged from ceiling at 7 feet above the ground level for proper lighting.

At the end of experiment, i.e. on the 42^nd^ day, blood was collected from the wing vein from four birds per treatment. Serum was separated out after centrifugation and stored at −20°C until further analysis. Hemolysate was prepared from plasma-separated blood and stored at −20°C. The requisite stored samples were evaluated for liver function test (glucose, total protein [TP], albumin [ALB], and globulin), lipid profile (cholesterol and triglyceride), and kidney function test (alanine aminotransferase [ALT], aspartate aminotransferase [AST], blood urea nitrogen [BUN], and creatinine) by using Erba (Mannheim) kits. Antioxidant levels (catalase, superoxide dismutase [SOD], lipid peroxidation [LPx], glutathione peroxidase [GPx], glutathione [GSH], and Vitamins E and C) were analyzed using standard methods.

### Statistical analysis

The collected data were analyzed using Statistical Package for the Social Sciences [10] at 95% significant level using Duncan’s level of significance values [11].

## Results

### Effect of CW

Supplementation of diet with CW, irrespective of supplementation of enzymes, showed no significant (p≤0.05) difference in glucose, TP, ALB, and globulin level (Table-1). However, the increased trend of blood glucose level was found with increased level of supplementation of diet with the highest glucose level (293.74 mg/dl) observed in 7.5% CW-supplemented diet and the lowest (267 mg/dl) was observed in control group.

**Table-1:** Effect of feeding different levels of citrus waste on the blood parameters of broiler.

Citrus waste (%)	Glucose (mg/dl)	TP (g/dl)	ALB (g/dl)	Globulin (g/dl)
0	267.24	2.68	1.67	1.01
2.50	272.86	2.62	1.72	0.90
5.00	289.54	2.70	1.74	0.96
7.50	293.74	2.74	1.72	1.02
Pooled SEM	14.053	0.114	0.077	0.085
p-value	0.602	0.705	0.711	0.520

TP=Total protein, ALB=Albumin, SEM=Standard error of mean

Supplementation of diet with CW showed no difference in the cholesterol levels; however, the level decreased with an increase in the level of supplementation (Table-2), irrespective of enzyme supplementation. Supplementation of diet with CW, irrespective of supplementation of enzyme cocktail, decreased the plasma triglyceride levels of birds (p≤0.05) at all levels of supplementation.

**Table-2:** Effect of feeding different levels of citrus waste on the lipid profile of blood in broiler.

Citrus waste (%)	Cholesterol (mg/dl)	Triglyceride (mg/dl)
0	109.70	96.66^a^
2.50	102.70	72.73^ab^
5.00	98.63	64.08^ab^
7.50	91.73	55.02^b^
Pooled SEM	6.647	11.737
p-value	0.509	0.041

a,b Means bearing different superscripts in a column differ significantly (p≤0.05). SEM=Standard error of mean

Creatinine levels and BUN were not affected by supplementation of CW, irrespective of supplementation of enzyme cocktail (Table-3). Supplementation of diet with CW decreased (p<0.05) the activities of AST and ALT linearly, irrespective of supplementation of enzymes. The AST level varied (p<0.05) from 134.99 IU/L (diet supplemented with 7.5% CW) to −165.4 IU/L (unsupplemented control diet), whereas ALT level varied (p<0.05) from 1.58 IU/L (birds fed diet supplemented with 5% CW) to 3.29 IU/L (birds fed unsupplemented control diet).

**Table-3:** Effect of feeding different levels of citrus waste on the liver and kidney function tests in broiler.

Citrus waste (%)	BUN (mg/dl)	Creatinine (mg/dl)	AST (IU/L)	ALT (IU/L)
0	3.18	0.37	165.74^a^	3.29^a^
2.50	3.91	0.36	148.20^ab^	2.22^ab^
5.00	3.34	0.56	144.57^b^	1.58^b^
7.50	3.33	0.47	134.99^b^	1.83^b^
Pooled SEM	0.43	0.096	6.127	0.454
p-value	0.610	0.703	0.025	0.038

a,b Means bearing different superscripts in a column differ significantly (p≤0.05). BUN=Blood urea nitrogen, ALT=Alanine aminotransferase, AST=Aspartate aminotransferase, SEM=Standard error of mean

Antioxidant status revealed that supplementation of diet with CW improved (p<0.05) the activities of SOD and LPx and these activities increased linearly with an increase in the level of supplementation of CW, irrespective of enzymes. The GPx, GSH, and Vitamins C and E were observed to be highest in birds fed diet supplemented with CW at 5%, and further increase decreased these parameters. Catalase activity differed non-significantly (Table-4).

**Table-4:** Effect of feeding different levels of citrus waste on the antioxidant status of blood in broiler.

Citrus Waste (%)	CAT (U/gHb)	SOD (U/mgHb)	LPx (nmol/gHb)	GPx (U/g Hb)	GSH (μg/ml)	Vitamin E (μmol/L)	Vitamin C (mg/dl)
0	0.10	18.48^b^	365.50^ab^	35.40^b^	8.18^b^	0.17^ab^	1.15^b^
2.50	0.09	22.82^ab^	348.50^b^	67.89^ab^	10.91^ab^	0.12^b^	1.09^b^
5.00	0.11	23.91^ab^	329.50^b^	108.14^a^	14.64^a^	0.29^a^	1.76^a^
7.50	0.14	32.61^a^	395.00^a^	40.72^b^	11.82^ab^	0.19^ab^	1.32^ab^
Pooled SEM	0.043	3.578	12.375	15.975	1.597	0.044	0.149
p-value	0.711	0.021	0.011	0.034	0.042	0.046	0.013

a,b Means bearing different superscripts in a column differ significantly (p≤0.05). CAT=Catalase, SOD=Superoxide dismutase, LPx=Lipid peroxidation, GPx=Glutathione peroxidase, GSH=Glutathione

### Effect of enzymes

Supplementation of CW-based diet with enzymes showed no significant difference in glucose, BUN, creatinine, TP, ALB, globulin, ALT, catalase activity, SOD activity, LPx activity, and GPx activity in blood, irrespective of levels of supplementation of CW in diet. However, triglyceride and cholesterol levels were higher (p≤0.05) in bird fed CW-based diet supplemented with enzymes in comparison to unsupplemented diet (Tables-5-8). Supplementation of CW-based diet with enzymes decreased (p<0.05) AST levels and ALT levels (p>0.05). GSH activity and Vitamin C in blood were observed in birds fed CW-based diet supplemented with enzymes; however, Vitamin E levels decreased on supplementation of enzymes.

**Table-5:** Effect of feeding different levels of enzymes on the blood parameters of broiler.

Enzymes (g/q)	Glucose (mg/dl)	TP (g/dl)	ALB (g/dl)	Globulin (g/dl)
0	279.57	2.58	1.64	0.94
100	282.11	2.80	1.79	1.01
Pooled SEM	9.937	0.081	0.054	0.06
p-value	0.806	0.809	0.708	0.725

TP=Total protein, SEM=Standard error of mean

**Table 6:** Effect of feeding different levels of enzymes on the lipid profile of blood in broiler.

Enzymes (g/q)	Cholesterol (mg/dl)	Triglyceride (mg/dl)
0	91.68^b^	58.10^b^
100	109.70^a^	86.15^a^
Pooled SEM	4.70	8.299
p-value	0.032	0.040

a,b Means bearing different superscripts in a column differ significantly (p≤0.05). SEM = Standard error of mean

**Table-7:** Effect of feeding different levels of citrus waste on the liver and kidney function tests in broiler.

Enzymes (g/q)	BUN (mg/dl)	Creatinine (mg/dl)	AST (IU/L)	ALT (IU/L)
0	3.67	0.48	156.90^a^	2.50
100	3.21	0.41	139.81^b^	1.96
Pooled SEM	0.304	0.068	4.333	0.321
p-value	0.520	0.562	0.021	0.504

a,b Means bearing different superscripts in a column differ significantly (p≤0.05). BUN=Blood urea nitrogen, ALT=Alanine aminotransferase, AST=Aspartate aminotransferase, SEM=Standard error of mean

**Table-8:** Effect of feeding different levels of enzymes on the antioxidant status of blood in broiler.

Enzymes (g/q)	CAT (U/gHb)	SOD (U/mgHb)	LPx (nmol/gHb)	GPx (U/g Hb)	GSH (μg/ml)	Vitamin E (μmol/L)	Vitamin C (mg/dl)
0	0.07	23.37	352.25	59.06	9.49^b^	0.26^a^	0.45^b^
100	0.15	25.54	367.00	67.01	13.29^a^	0.13^b^	2.22^a^
Pooled SEM	0.03	2.53	8.751	11.296	1.13	0.031	0.105
p-value	0.801	0.705	0.607	0.708	0.045	0.012	0.023

a,b Means bearing different superscripts in a column differ significantly (p≤0.05). CAT=Catalase, SOD=Superoxide dismutase, LPx=Lipid peroxidation, GPx=Glutathione peroxidase, GSH=Glutathione, SEM=Standard error of mean

### CW × enzyme

Data were analyzed to see the interactions between levels of supplementation of CW and inclusion of enzymes in CW-based diet, and the results revealed that TP varied (p<0.05) from 2.16 g/dl (birds fed diet supplemented with CW at 2.5%) to 3.09 g/dl (birds fed diet supplemented with CW at 2.5% and enzymes). Serum albumin and globulin followed the trend of TP (Table-9). Supplementation of CW-based diet with enzymes decreased (p<0.05) blood cholesterol levels, whereas the serum TG levels increased when CW-based diet was supplemented with enzymes (Table-10). Supplementation of diet with CW decreased (p<0.05) AST and addition of enzymes to CW-based diet further lowered (p<0.05) the AST activity. A similar trend was observed for ALT (Table-11).

**Table-9:** Effect of citrus waste×enzyme on the blood parameters of broiler.

Treatments	Citrus (%)	Enzymes (g/q)	Glucose (mg/dl)	TP (g/dl)	ALB (g/dl)	Globulin (g/dl)
C	0	0	269.56	2.75^a^	1.68^ab^	1.07^a^
CW	2.50	0	288.26	2.16^b^	1.54^b^	0.62^b^
CW	5.00	0	258.08	2.69^a^	1.67^ab^	1.01^a^
CW	7.50	0	302.39	2.72^a^	1.67^ab^	1.05^a^
CE	0.00	100	264.91	2.62^ab^	1.66^ab^	0.95^ab^
CWE	2.50	100	299.22	3.09^a^	1.91^a^	1.18^a^
CWE	5.00	100	287.63	2.72^a^	1.82^ab^	0.90^ab^
CWE	7.50	100	276.69	2.76^a^	1.76^ab^	1.00^a^
Pooled SEM			19.874	0.162	0.108	0.12
p-value			0.602	0.042	0.031	0.011

a,b Means bearing different superscripts in a column differ significant (p≤0.05). TP=Total protein, ALB=Albumin, SEM=Standard error of mean

**Table-10:** Effect of citrus waste×enzyme on the lipid profile of blood in broiler.

Treatment	Citrus waste (%)	Enzymes (g/q)	Cholesterol (mg/dl)	Triglyceride (mg/dl)
C	0	0	119.45^a^	65.56^b^
CW	2.50	0	114.28^ab^	62.94^sb^
CW	5.00	0	106.40^b^	60.94^b^
CW	7.50	0	98.68^b^	42.96^c^
CE	0.00	100	105.13^ab^	127.76^a^
CWE	2.50	100	90.85^ab^	84.52^a^
CWE	5.00	100	85.95^b^	67.09^b^
CWE	7.50	100	84.78^b^	65.23^b^
Pooled SEM			9.40	16.598
p-value			0.021	0.045

a,b Means bearing different superscripts in a column differ significantly (p≤0.05). SEM=Standard error of mean

**Table-11:** Effect of citrus waste × enzyme on the liver and kidney function tests in broiler.

Treatment	Citrus waste (%)	Enzyme (g/dl)	BUN (mg/dl)	Creatinine (mg/dl)	AST (IU/L)	ALT (IU/L)
C	0	0	3.35	0.37	182.60^a^	3.16^a^
CW	2.50	0	4.33	0.35	160.08^b^	2.39^b^
CW	5.00	0	4.18	0.78	154.05^bc^	2.37^b^
CW	7.50	0	2.83	0.41	130.87^c^	2.08^b^
CE	0	100	3.00	0.37	148.89^bc^	3.43^a^
CWE	2.50	100	3.50	0.38	138.95^bc^	2.37^b^
CWE	5.00	100	2.50	0.35	136.33^bc^	1.30^c^
CWE	7.50	100	3.83	0.53	135.09^bc^	0.77^d^
Pooled SEM			0.609	0.135	8.665	0.642
p-value			0.804	0.807	0.011	0.031

a,b Means bearing different superscripts in a column differ significantly (p≤0.05). BUN=Blood urea nitrogen, ALT=Alanine aminotransferase, AST=Aspartate aminotransferase, SEM=Standard error of mean

Data on antioxidant status revealed that LPx activity varied from 309 nmol/gHb (birds fed diet supplemented with 5% CW) to 425 mmol/gHb (birds fed CW [at 7.5%]-based diet supplemented with enzymes). GPx and GSH activities and serum Vitamin C levels were observed to be highest (p<0.05) in birds fed CW (at 5%)-based diet supplemented with enzymes (Table-12). Birds fed diet supplemented with CW at 5% had the highest (p<0.05) levels of Vitamin E, and the addition of enzymes in CW-based diets decreased Vitamin E levels in blood. However, the catalase and SOD activities showed no significant difference between the groups.

**Table-12:** Effect of citrus waste × enzyme on the antioxidant status of blood in broiler.

Treatment	Citrus waste (%)	Enzyme (g/dl)	CAT (U/gHb)	SOD (U/mgHb)	LPx (nmol/gHb)	GPx (U/g Hb)	GSH (μg/ml)	Vitamin E (μmol/L)	Vitamin C (mg/dl)
C	0	0	0.04	19.57	392.00^ab^	33.67^c^	9.29^bc^	0.19^bc^	0.31^c^
CW	2.50	0	0.05	17.39	343.00^b^	77.27^b^	9.37^bc^	0.08^c^	0.42^c^
CW	5.00	0	0.09	23.91	309.00^c^	96.98^a^	10.02^bc^	0.44^a^	0.58^c^
CW	7.50	0	0.10	32.61	365.00^b^	28.32^c^	9.25^bc^	0.33^ab^	0.48^c^
CE	0.00	100	0.08	17.39	339.00^b^	37.12^c^	7.07^c^	0.15^bc^	1.87^b^
CWE	2.50	100	0.16	23.91	354.00^b^	58.50^b^	12.45^b^	0.15^bc^	1.89*^β^*
CWE	5.00	100	0.18	28.26	350.00^b^	119.30^a^	20.03^a^	0.14^bc^	2.94^a^
CWE	7.50	100	0.18	32.61	425.00^a^	53.12^b^	13.62^b^	0.06^c^	2.16^b^
Pooled SEM			0.061	5.06	17.501	22.592	2.259	0.063	0.211
p-value			0.708	0.809	0.021	0.011	0.039	0.044	0.038

a,b,c Means bearing different superscripts in a column differ significantly (p≤0.05). SOD=Superoxide dismutase, LPx=Lipid peroxidation, GPx=Glutathione peroxidase, GSH=Glutathione, SEM=Standard error of mean

## Discussion

### Effect of CW

Citrus pulp supplementation at the level of 6% in broiler ration did not show any negative effect on blood glucose level [12]. TP, ALB, and globulin did not vary significantly and were found within normal range [13]. It was also reported that inclusion of orange peel extract (OPE) and lemon peel extract (LPE) did not influence TP, ALB, and globulin [14] in blood. The cholesterol and triglyceride levels of blood decreased (p≤0.05) with an increase in the levels of CW in the diet [6,15-17]. The reduced cholesterol level of the blood could have been contributed by the pectin present in the citrus. It has been reported that pectin reduced pancreas enzyme activity, which resulted in increased fecal fat excretion [18] and lower fat deposition. ALT, AST, BUN, and creatinine levels were found to be in normal range [13] indicating normal functioning of the liver and kidney. It was also found that inclusion of higher levels of LPE, OPE, and *Curcuma xanthorrhiza* essential oil reduced the AST activity (p≤0.05) without having any effect on BUN and creatinine level of blood [14]. The antoxidant levels i.e. SOD, catalase and GPx in the broiler blood increased with increase in levels of CW in the feed [19]. Supplementation of diet with LPE resulted in higher level of GPx activity (p≤0.05) in birds [14].

### Effect of enzymes

It was found that there was a decrease in the level of blood cholesterol and triglyceride levels of blood (p≤0.05) where orange waste without and with enzymes fed to chicken [15]. Lower cholesterol and triglyceride levels in the blood could be due to the presence of hesperidin in the CW.

### CW × enzyme

Supplementation of diet with dried lemon pulp in broiler ration showed no significant difference in the glucose, TP, ALB, and globulin level of the blood [4]. Citrus pulp supplemented at the level of 6% in broiler ration showed lowest blood cholesterol and triglyceride level [12]. Diet supplemented with sweet orange and other citrus fruits to broilers was efficient in lowering lipid profile of the blood [20,21]. It has been observed that increased level of CW in the broiler diet increased blood antioxidant status [19]. Citrus by-products are good source of Vitamin C [16] and contain substance showing antioxidant activity that is attributable to the flavones, i.e., hesperidins.

## Conclusion

The blood profile showed that supplementation of CW up to 5% decreased cholesterol, triglyceride, and AST levels and improved the antioxidant status without affecting TP, ALB, globulin, and BUN levels. Vitamin C levels were observed to be highest (p<0.05) in birds fed CW (at 5%)-based diet supplemented with enzymes.

## Authors’ Contributions

DPB conducted the experiment and wrote the manuscript. CS helped in processing of samples in Veterinary Biochemistry Laboratory. APSS helped in statistical analysis of the results obtained. APSS and US helped in nutritional and managemental aspect of this study. MW helped in planning and execution of the work. All authors contributed in drafting the manuscript. All authors corrected the manuscript and read and approved the final manuscript.
